# Design of Surgical Impaction Instruments Matters

**DOI:** 10.1016/j.artd.2025.101898

**Published:** 2025-11-12

**Authors:** Peter J. Schlieker, Frank Lampe, Johann Zwirner, Benjamin Ondruschka, Michael M. Morlock, Gerd Huber

**Affiliations:** a Institute of Biomechanics, Hamburg University of Technology, Hamburg, Germany; b Department of Orthopaedics, Asklepios Klinik Barmbek, Hamburg, Germany; c Faculty of Life Sciences, Hamburg University of Applied Sciences, Hamburg, Germany; d Institute of Legal Medicine, University Medical Center Hamburg-Eppendorf, Hamburg, Germany; e Department of Oral Sciences, University of Otago, Dunedin, New Zealand

**Keywords:** Total hip arthroplasty, Force transmission, Impaction, Cadaveric study, Numerical simulation

## Abstract

**Background:**

Femoral stem impaction in total hip arthroplasty is commonly performed by mallet blows on a metal impactor attached to the stem. Factors including the surgeon, the impactor, and the patient can influence the impaction. A wide range of impactors, varying in design and thus in mass and stiffness, are available. However, little is known about their influence on the force transmission and, consequently, about the proportion of the mallet force that ultimately reaches the implant. This study aimed to investigate the force transmission through the impactor for different impactor designs, while investigating different patient-specific femur-tissue systems in situ and in silico.

**Methods:**

The mallet and impactor forces of 9 consecutive blows on seated femoral stems were measured for 2 different approaches on each of 4 cadavers. The mallet-implant force transmission was calculated using a phenomenological model for 2 different impactor designs.

**Results:**

The attenuated force in the impactor achieved approximately 65% to 75% of the corresponding mallet peak force, regardless of cadaver or surgical approach. Measuring the force distant from the tip resulted in an overestimation of the transferred forces. Depending on impactor design just 24% to 47% of the applied mallet peak force reached the implant itself.

**Conclusions:**

The force transmission for overcritical mallet blows can be regarded as independent from patient- and approach-specific boundary conditions and primarily dependent on the impactor design. Surgeons must be aware of this relationship and exercise caution when using other or novel instruments to prevent intraoperative complications.

## Introduction

In total hip arthroplasty (THA), cementless stems are held through press-fit in the undersized femoral cavity. The required impaction force is applied via mallet blows on a metal impactor. Intraoperatively, the surgeon must achieve a trade-off between inadequate press-fit of undersized stems—bearing the risk of aseptic loosening [1,2]—and excessive implantation forces caused by impacting an oversized implant and increase the risk of a peri-prosthetic fracture [1,3,4].

This procedure is far from being standardized, as these blows are known to vary not only between surgeons [5,6], but also between surgeon’s practice [[7], [8], [9]]. Since many surgeons have different preferences regarding their mallets [10], these cannot be standardized either. While little is known about the influence of the impactor on the force transmission [11,12], the impactor design exhibits large variability, with options including solid or slotted handles and straight or curved shafts with different diameters and lengths [11]. Since the force is transmitted from the mallet to the stem, the blow traverses the impactor and likely undergoes impactor-specific attenuation [[11], [12], [13]]. In case this force transmission could be improved, the requisite applied force by the mallet could be reduced, while the same amount of force reaches the stem. This could have beneficial effects on the health of the surgeons [14,15]. However, due to the dynamic characteristics of the mallet blows applied during THA, the force transmission cannot be examined independently for each part along the transmission path. The force transmission inside the impactor may be influenced by other components, including the implant or the patient’s body. Whether the flexibility of the femur-tissue system (FTS) needs to be considered during the impaction of stems is discussed controversially in the literature. While the majority of in vitro studies continue to use rigid mountings for their specimens [1,[16], [17], [18], [19], [20], [21], [22]], some studies included soft boundary conditions in their test setups [9,12,[23], [24], [25]] and Doyle et al. was the first to quantify the parameters for future in vitro testing [26]. However, a recent cadaveric study concluded that metal-on-metal impacts would be overcritical for the rather soft FTS [27] and would therefore not affect the force transmission from the mallet to the stem.

The aim of this study was, therefore, to examine whether differences in the design of the impactor result in different impaction forces at the stem in silico based on in situ cadaveric measurements.

## Material and methods

### Cadaveric measurement

Four fresh human cadavers, stored at 4 °C prior to the measurements, were part of this study (approved by the Ethics Commission of the Medical Association Hamburg (2024-300436-WF)). Besides sex (3 males, 1 female), age at death (35-65 years), weight (70.4 kg to 117.6 kg), and height (1.81 m to 1.84 m), all personal information was anonymized.

**Figure 1:**
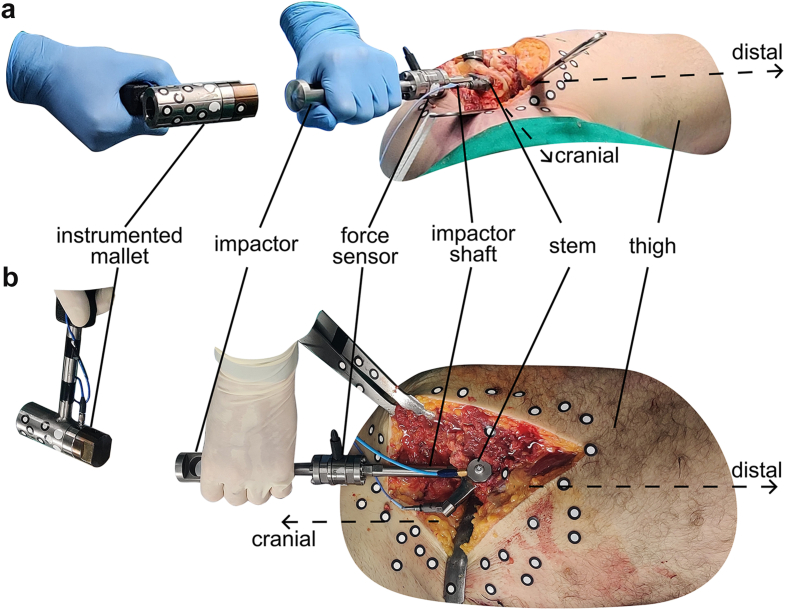
Measurement setup during (a) a lateral transgluteal approach and (b) a direct anterior approach. The additional sensors on the stem and femur and the optical markers were not related to this research question and did not interfere with the measurements. To guarantee the anonymity of the deceased, a visible tattoo was removed from one of the pictures.

After the rigor mortis was broken by mobilization of the hip, 2 THA surgeries were performed on each cadaver. One side was operated in lateral position with a lateral transgluteal approach [28] and the contralateral side was operated in supine position with a direct anterior approach. The assignment of the approaches to the sides was randomized for each cadaver. Both procedures were performed on the same day, with only one cadaver being operated per day. For the lateral transgluteal approach a standard tunnel pad and a set of lateral hip positioners were used to stabilize the cadavers. All steps from incision to stem implantation were performed by the same experienced surgeon following the procedures of established THA surgeries. Impactions for broaching and implantation were applied with an automated surgical impaction tool (Kincise, Johnson & Johnson MedTech, Raynham, MA). The implanted stems were uncemented collarless tapered wedge stems (Corail, Johnson & Johnson MedTech, Raynham, MA). The implantation was stopped when the experienced surgeon considered the stem as fixed within the femur without detectable movement. When fully seated, 10 additional manual blows were applied to the stem using an instrumented mallet with a piezo force sensor at the tip (Fig. 1; total weight 890 g, 9041A, Kistler, Winterthur, CH) and an instrumented impactor. The instrumented impactor was similar to the original stem impactor belonging to the implant system, but modified in a way to allow the mounting of a force sensor (9333A, Kistler, Winterthur, CH) with 2 flange connections. The surgeon could choose between 2 different lengths of the impactor shaft below the force sensor (50 mm and 100 mm) to enable access to the stem through different thicknesses of soft tissue without pushing the sensor too deep into the surrounding tissue. The first blow for each approach on each cadaver served as preconditioning to achieve proper contact between the impactor tip and the stem. For the 9 consecutive mallet blows of each of the 8 implantations, forces at the tip of the mallet and in the shaft of the impactor were recorded with 500 kHz and 14 bits (NI-9775, National Instruments, Austin, TX).

### Data evaluation

The measured force signals (n = 72) of the mallet and the impactor were filtered with a fourth-order zero-phase low-pass filter with a cut-off frequency of 24 kHz to address the oscillating mass of the mallet tip on the stiffness of the corresponding force sensor. Then, the maximum mallet and impactor forces were determined. To account for the variability of manually applied mallet blows, the impactor forces were normalized by the corresponding maximum of the mallet force. This dimensionless ratio of the force at the impactor to the maximum force of the mallet is the attenuation of the respective force transmission from one sensor position to another [13].

The measured force transmission was further evaluated with a transfer function in the frequency domain. Therefore, the discrete Fourier transform was calculated for the unfiltered force signals from both sensor positions (Matlab R2024b, MathWorks, Natick, MA). To improve the signal to noise ratio, the mallet force was windowed with a narrow rectangular window [29] and both forces were windowed with an exponential window including compensation with its coherent gain [[30], [31], [32]]. For the resulting transfer function, which is the ratio of output force (impactor) to input force (mallet) in the frequency domain, values greater than 1 indicate amplification, whereas values less than 1 correspond to attenuation for the corresponding frequency—likewise the force ratio mentioned above. Based on the consecutive blows, the averaged transfer function was generated by calculating the median and the interquartile ranges (IQRs) for every frequency.

### Phenomenological model

**Figure 2:**
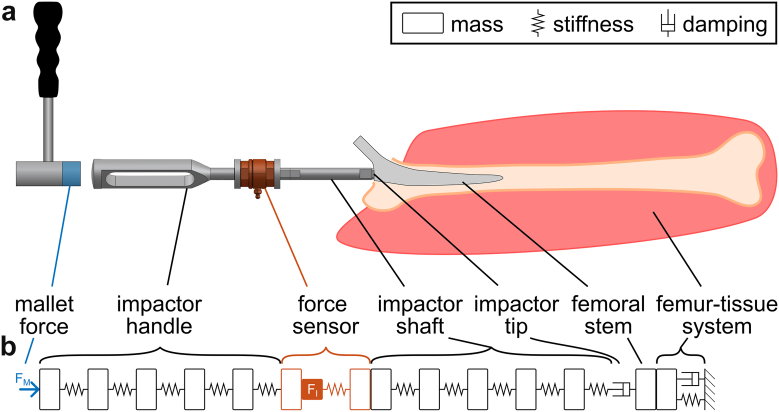
(a) Structure of a simplified version of the experimental setup and (b) the corresponding structure of the phenomenological model.

The setup of the cadaveric measurements was rebuilt with a phenomenological model (Fig. 2; Table 1; Simscape, Simulink R2024b, MathWorks) based on differential equations. The model was based on a previously validated model [33] and only a few adaptations were required to adjust it to the current study: The impactor had a more complex design than in the original study [13] and therefore the handle and the shaft were each divided into 5 single degree of freedom mass-spring systems in series to account for different segments with different masses, cross sections and stiffnesses. The respective masses were obtained from a 3D computer-aided design model (SolidWorks 2020 SP5.0, Dassault Systèmes, FR). The corresponding stiffnesses of the impactor were calculated using a finite element analysis (SolidWorks 2020 SP5.0). In the phenomenological model, the mass of the force sensor in the impactor was divided in half and implemented above and below its stiffness. The interface between the impactor tip and the implant was simulated by a simplified approach using a translational damper that represented the plastic deformation of the impactor tip observed during the cadaveric measurements. Moreover, this damper covered all the other damping effects of the surgical instrument. The stem was included as a single mass and rigidly constrained to the limb, since no relative movement between the stem and the femur was expected after fully seating of the stem with the automated surgical impaction tool. The parameters of the single degree mass-spring-damper system of the FTS were updated with the values determined for the same cadavers and approaches [27]. The simulations were performed with a constant step size of 0.5 μs, but the results were resampled according the acquisition rate during the measurements. Each measured mallet blow applied by the surgeon was used as input signal to numerically determine the corresponding impactor force and the resulting attenuation identically as for the measured data. The accuracy of the model was evaluated by comparing simulated and measured forces. To address the issue of potential phase shifts, Dynamic Time Warping [34] was applied, aligning the signals with nonlinear time adjustments. A small normalized warping path length close to 1 is indicative of a high degree of similarity between the 2 force signals, while an increased distance represents a greater disparity. The root mean squared error between measured and simulated normalized impactor forces was calculated for the initial 2 ms of the warped signals. This approach provides an effective measure of alignment accuracy while accounting for temporal discrepancies. The transfer function of the model was calculated between the force input and the force output at the impactor.

**Table 1:** Parameters of the phenomenological model for the short impactor shaft (50 mm).

Object	Mass [kg]	Stiffness [N/mm]	Damping [Ns/m]
Impactor handle			
Part 1	0.049	3.968 × 10^6^	
Part 2	0.109	0.415 × 10^6^	
Part 3	0.039	0.491 × 10^6^	
Part 4	0.021	0.745 × 10^6^	
Part 5	0.032	6.266 × 10^6^	
Force sensor	2 × 0.069	2.3 × 10^6^	
Impactor shaft			
Part 1	0.024	30.303 × 10^6^	
Part 2	0.017	1.015 × 10^6^	
Part 3	0.009	2.331 × 10^6^	
Part 4	0.008	1.238 × 10^6^	
Part 5	0.001	2.739 × 10^6^	
Impactor tip			1500
Femoral stem	0.094-0.123		
FTS	10.85-17.86	24.23-49.65	312.0-785.9

The parts 1-5 of the impactor handle and the impactor shaft correspond to the 2 sets of 5 single degree of freedom mass-spring systems in series that were required to describe the complex geometry of the surgical instrument.

**Figure 3:**
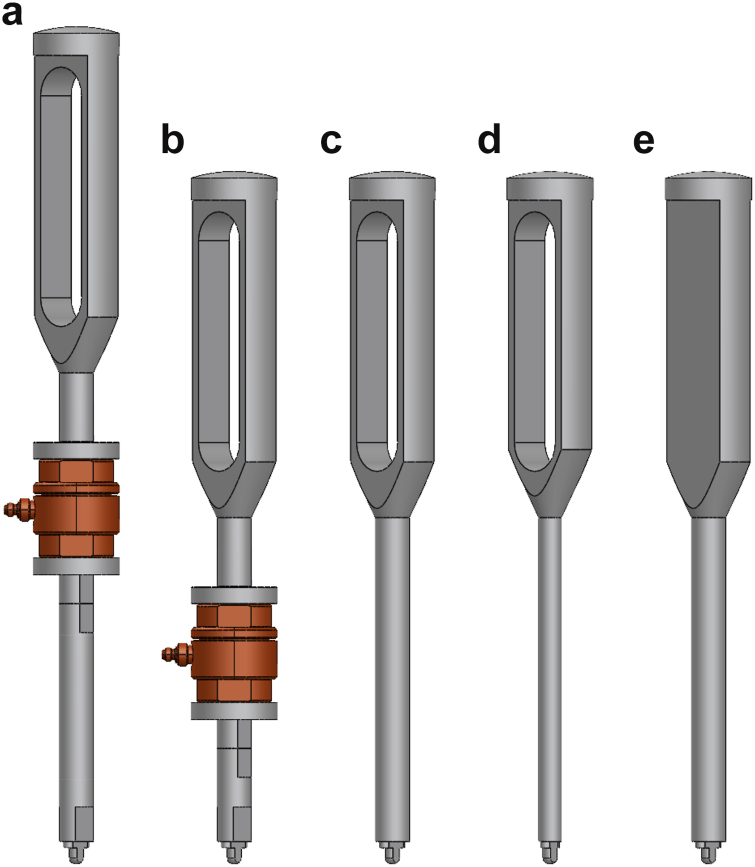
Overview of the different impactors used in the simulations: (a) thick and long shaft with force sensor and slotted handle, (b) thick and short shaft with force sensor and slotted handle, (c) thick shaft with slotted handle (‘reference’), (d) slim shaft with slotted handle (‘slim’), and (e) thick shaft with solid handle (‘solid’).

However, the phenomenological model was specially used to determine the force at the distal tip of the impactor. Attaining this value through measurements is challenging, but given its role as the point of final force transfer from the implantation instruments to the femoral stem, it is in fact the principal point of interest. Moreover, the masses and stiffness of the force sensor were only included in the model (Fig. 3a and b) to replicate the measurements as closely as possible but the sensor is not of clinical relevance. Therefore, to investigate how the utilization of a sensor might affect the acting forces, the sensor was replaced by a cylindrical component of the shaft in subsequent simulations (Fig. 3c; ‘reference’). The impact of the impactor design was evaluated by simulating 2 realistic variations of the reference impactor: a slim version with an 8 mm thick shaft and a slotted handle (Fig. 3d; ‘slim’) and a solid version with a 12 mm thick shaft and a solid handle (Fig. 3e; ‘solid’).

### Statistical analysis

The statistical analysis of the force attenuation was conducted using a type I error level of α = 0.05 (SPSS 29.0, IBM, Armonk, NY). The data were tested for normal distribution and homogeneity of variance using the Shapiro–Wilk test and the Levene’s test, respectively. Even though the approaches could be considered as dependent conditions, since they were performed on both sides of each cadaver, the nonordered sequence of blows within each approach led to an independent test condition. Comparisons of the approaches were performed with the Mann–Whitney U test. The cadavers, the different evaluation positions and the different impactor designs were compared with the Kruskal–Wallis test including Bonferroni correction as post-hoc analysis. In case of a nonsignificant result, a power analysis for the difference between the approaches was performed using a statistical power of 1-β = 0.8 [35]. Boxplots with whiskers up to 1.5 IQR in length were used to visualize nonparametric results. Values outside this range were defined and highlighted as outliers but not excluded from the statistical analysis.

## Results

### Cadaveric measurement

**Figure 4:**
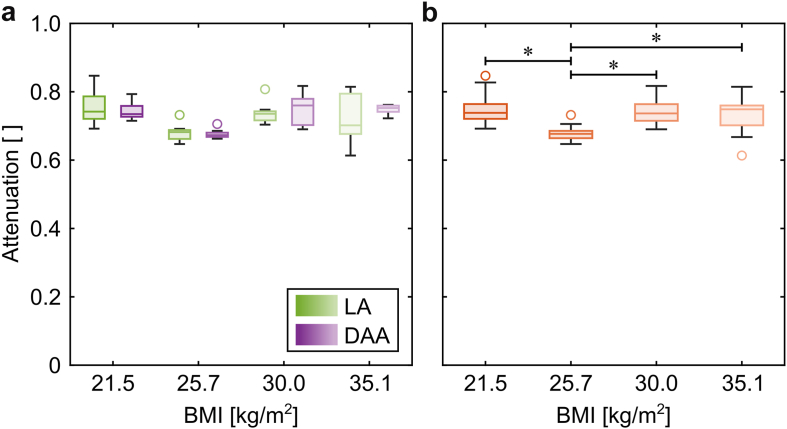
Force attenuation from the mallet to the impactor for each of the 4 cadavers ordered by their increasing BMI. (a) No significant differences occurred between the lateral transgluteal approach and the direct anterior approach for each of the cadavers. (b) When the approaches for each cadaver were combined, the attenuation for the cadaver with a BMI of 25.7 kg/m^2^, which was the one on which the shorter impactor was used, was lower than for the other 3. BMI, body mass index; DAA, direct anterior approach; LA, lateral transgluteal approach.

In 3 cadavers, the surgeon selected the longer impactor shaft for both approaches, while in the remaining cadaver, he used the shorter impactor shaft on both sides. The maximum forces of the mallet blows ranged from 7.7 kN to 23.9 kN, resulting in forces ranging from 5.6 kN to 16.0 kN for the impactor. The attenuation, received by the normalization, was similar for the 2 approaches of each of the cadavers (Fig. 4a; 21.5 kg/m^2^: *P* = .796, 25.7 kg/m^2^: *P* = .730, 30.0 kg/m^2^: *P* = .863, 35.1 kg/m^2^: *P* = .340). Consequently, the attenuation could be combined for each of the cadavers. The normalized peak forces at the impactor were lower for one cadaver (Fig. 4b; all *P* < .001), while did not differ for the remaining 3 (all remaining *P* = 1.000). This coincided with the usage of the shorter impactor shaft for this particular cadaver. Thus, the measurements resulted in different attenuations for the long shaft (median 73.9%, IQR 71.7%-76.2%) and the short shaft (median 67.7%, IQR 66.4%-68.6%). With the given distribution, the 0.7% difference in force transmission between the 2 approaches could only deliver statistical significance for a sample size of approximately 600 cadavers.

### Simulation

**Figure 5:**
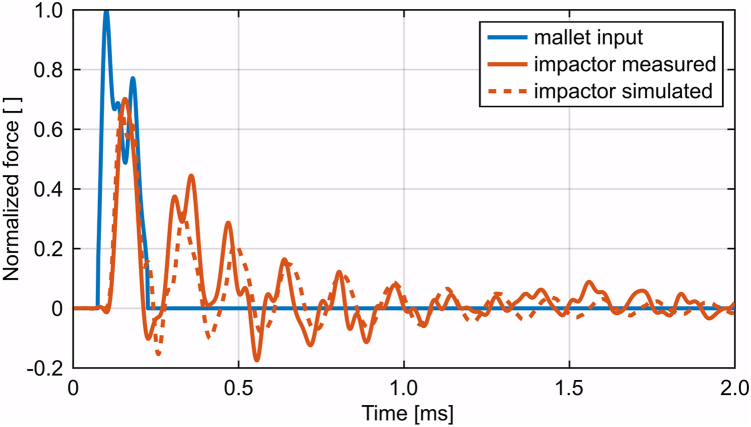
Exemplary simulation result of a mallet blow on the long impactor in comparison to the corresponding measured attenuation. The harmonic oscillation of the mallet tip after the main peak was removed for the simulation input since it was caused by a vibration of the tip and was not part of the force transfer between the mallet and the impactor.

The exponential decay of the oscillating force of the impactor could approximately be reproduced by the simulation (Fig. 5). An underestimation of the peak force of about 7.5% to 10% of the corresponding mallet peak force remained. However, the model could explain the pronounced attenuation for the shorter impactor (*P* < .001; long: 66.4%, IQR 65.1%-69.0%; short: 57.7%, IQR 56.8%-59.0%). The normalized median warping path length was 1.54 (IQR 1.51-1.57) resulting in a median root mean squared error of 3.2% (IQR 2.8%-3.9%) for the aligned impactor forces.

### Transfer function

**Figure 6:**
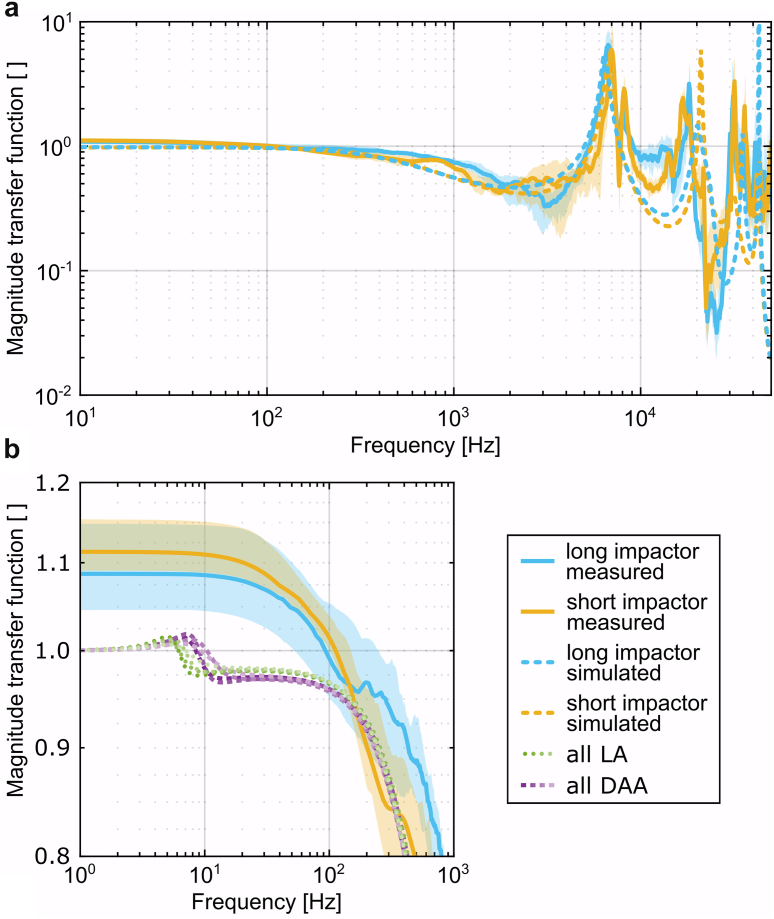
(a) Transfer functions for the long impactor (combination of 54 blows), the short impactor (combination of 18 blows) and the corresponding models. (b) Transfer functions of the model for the different parameters of the femur-tissue system [27] together with the transfer functions for the measurements with a focus on a lower frequency range and a strong magnification of the magnitude. The solid lines represent the medians, while the shaded areas indicate the IQRs (25th-75th percentiles). DAA, direct anterior approach; LA, lateral transgluteal approach.

The transfer functions of the measurements showed comparable progressions and were pooled for each of the impactor lengths (Fig. 6a). The 2 groups resulted in similar curves with only minor deviations in magnitude and in areas with extinct attenuation (frequencies with little load transfer). The transfer function of the model generally followed a very similar shape like the measurements (Fig. 6a). The modified mass-spring system of the impactor shaft for the 2 shaft lengths led to some changes in the transfer function that were predominantly similar to those measured. For low frequencies close to the eigenfrequency of the FTS (around 10 Hz), only minor differences between the various FTS were observed in the transfer function of the model (Fig. 6b). For high frequencies above 40 kHz the simulation failed to represent the measurements.

### Impactor variation

**Figure 7:**
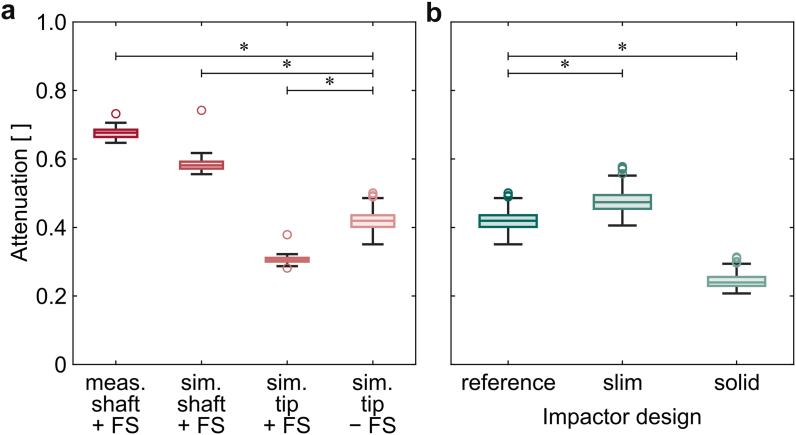
(a) Measured (meas.) and simulated (sim.) attenuation for the sensor position and the distal tip of the short impactor with and without force sensor (+/− FS) and (b) simulated attenuation to the impactor tip for the reference, slim, and solid impactor design. The simulated attenuation to the distal tip of the short impactor without a force sensor is the same as for the reference impactor design. FS, force sensor.

The calculated force at the tip of the impactor was clearly lower than that measured at the sensor position in the middle of the shaft (Fig. 7a; *P* < .001). 58.1% (IQR 57.2%-59.2%) of the mallet peak force reached the shaft and 30.6% (IQR 30.0%-31.1%) the tip, respectively. The removal of the force sensor resulted in higher forces at the tip of the impactor, with 42.0% (IQR 40.2%-43.6%; *P* < .001) of the corresponding mallet peak force. Regarding the different designs of the impactor, it is noteworthy that the slim impactor enabled the transfer of higher peak forces in comparison to the reference (Fig. 7b; all *P* < .001; median 47.4%, IQR 45.5%-49.5%), while the simulation for the solid impactor resulted in substantially lower peak forces (*P* < .001; median 24.0%, IQR 22.9%-25.6%).

## Discussion

The findings emphasized that the force transmission through the impactor remains unaffected by the patient- or approach-specific boundary conditions. Instead, changes of the impactor parameters had a strong influence on it. Relatively minor but yet realistic design changes resulted in a twofold increase in the transmitted peak force. A solid, and therefore heavier, impactor resulted in lower transmitted peak forces and requires higher applied forces to reach the same force at its tip.

The observed variation of mallet blows was within the reported range that can be found in the literature for impactions at the femoral side in cadaveric experiments [7,8].

The measured attenuations in this study differed considerably from the earlier described ones (35% [13] vs 74%), but this could firstly be attributed to the lower mass of the FTS in the other study and secondly to the usage of different impactors. These impactors varied not only in terms of their mass and stiffness of the metal components, but the head impactor also contained a polyether ether ketone tip, which was a major contributor to the attenuation observed in the previous study.

For the development of new implantation tools, differences in FTS can be disregarded as long as overcritical blows are applied. In case completely new approaches are investigated (eg, quasistatic pressing-in), the FTS might have to be considered to avoid tissue damages.

In the literature, lower stiffnesses of head impactor tips were linked to lower forces [12]. However, the opposite was observed in this study. It should be noted that the lower stiffness of the impactor shaft was accompanied by a relevant reduction in mass (0.100 kg vs 0.045 kg), while its stiffness was still orders of magnitude above the stiffness reported in the study, which revealed decreased forces by softer tips (84 × 10^3^ N/mm vs 34 N/mm to 11 × 10^3^ N/mm [12]).

Impaction forces cannot be measured without introducing sensors into the path of force transmission. Piezoelectric sensors have high stiffnesses that might not severely influence the dynamic behavior, but their mass will. This observer effect could be corrected within the phenomenological model—removing the sensor’s weight from the impactor increased the transmitted forces. Beside the influence of the sensor’s mass, the position of the force evaluation had a large influence [13]. As a consequence, future in situ and in vitro studies should select force sensors with minimal mass to reduce their effect on dynamics and select the position of measurement as close as possible to the position of interest, which can be very challenging when forces at interfaces are of interest. Alternatively, the presented model could be used to account for evaluation positions and force sensors.

Any kind of impactor, introducer, or broach handle will attenuate the force, but the degree can be manipulated by the design and the material of the instrument [12,13,36,37].

Certain limitations of the present study need to be addressed. An additional investigation was conducted on the same cadavers [27], which is why the incisions made were slightly longer than those typically performed in primary THA surgery. This had the advantage that the respective parameters of the mass-spring-damper model of the FTS were available.

The amount of force the surgeon applied to the impactor by pressing it toward the stem, and the damping effects of the surgeon’s hand or the tissue pressed against the impactor shaft were neglected. The model exclusively incorporated damping at the impactor tip, which likely led to an overestimation of the plastic deformation of the tip. This approach was a simplification that proved adequate for simulating very short impacts, but it needs to be reconsidered for longer simulation durations. The absence of additional dampers in the model was likely to cause the discrepancy in the transfer functions above 40 kHz. The model overestimates high-frequency vibrations; however, these frequencies are barely included in common metal-on-metal mallet blows and can therefore be neglected for the real-life impaction of femoral stems.

The analysis was performed on fully seated stems and it might be hypothesized that the stem–femur interface exhibits lower stiffness at the beginning of the seating process [7,24,38]. Nevertheless, the measured and simulated attenuations indicated that the FTS was of minor importance on the force transmission through the instruments since the impulses were far too short to excite the FTS with its low natural frequency [39].

Dividing the impactor into a series of single degree of freedom mass-spring systems was a simplification of the complex design of the surgical instrument, but the model received good agreement for the transfer function and especially for the force attenuation in the time domain.

## Conclusions

Changes in the design of the impactor have the potential to empower surgeons who might otherwise not be able to reach the desired stem position during THA. However, it could also lead to an increased risk of fracture if surgeons apply the same forceful blows as usual without taking the (new) impactor into consideration. Consequently, alterations to the impactor design must be approached with caution, and changed mechanical behavior must be widely elaborated to surgeons by the manufacturers to prevent intraoperative complications when new instruments are introduced.

## Conflicts of interest

Peter J. Schlieker receives institutional support by Johnson & Johnson MedTech. Benjamin Ondruschka is in the editorial board of “Rechtsmedizin” and “Notaufnahme up2date” and is a board member of the German Society of Legal Medicine. Michael M. Morlock received speakers bureau/paid presentations for Johnson & Johnson MedTech, Peter Brehm, Kyocera, Link, Mathys, and Enovis; is a paid consultant for Johnson & Johnson MedTech and Kyocera; and received research support as a Principal Investigator from Johnson & Johnson MedTech and Peter Brehm. Gerd Huber received institutional support as a Principal Investigator from Johnson & Johnson MedTech, Peter Brehm, and Link and is the president of the German Society of Biomechanics. The other authors declare no potential conflicts of interest.

For full disclosure statements refer to https://doi.org/10.1016/j.artd.2025.101898.

## CRediT authorship contribution statement

**Peter J. Schlieker:** Writing – original draft, Visualization, Software, Project administration, Methodology, Investigation, Formal analysis, Data curation, Conceptualization. **Frank Lampe:** Writing – review & editing, Investigation. **Johann Zwirner:** Writing – review & editing, Investigation. **Benjamin Ondruschka:** Writing – review & editing, Supervision, Resources. **Michael M. Morlock:** Writing – review & editing, Supervision, Resources, Funding acquisition. **Gerd Huber:** Writing – review & editing, Supervision, Resources, Methodology, Funding acquisition, Formal analysis, Conceptualization.

## Appendix A Supplementary data

Conflict of Interest Statement for All Authors
